#  Immune Infiltration Correlates with Transcriptomic Subtypes in Primary ER+ Invasive Lobular Breast Cancer 

**DOI:** 10.21203/rs.3.rs-4579052/v1

**Published:** 2024-08-13

**Authors:** Fangyuan Chen, Sayali Onkar, Jian Zou, Yujia Li, Haley Arbore, Sai Maley, George Tseng, Peter Lucas, Tullia Bruno, Dario Vignali, Julia Foldi, Marija Balic, Adrian Lee, Steffi Oesterreich

## Abstract

Understanding interplay of breast cancer and microenvironment is critical. Here, we identified two transcriptomic subtypes and five immune infiltration patterns from RNA-seq and multiplex immunohistochemistry from 21 ER+/HER2- invasive lobular breast cancers. The proliferative subtype associated with increased immune infiltration especially by immunosuppressive regulatory T-cells and macrophages. We also defined a TAM-Low signature, which associated with lower infiltration of proliferative, pro-inflammatory TAM, and improved outcome in patients with ER+ tumors.

## Main text

Immunotherapy (IO) is becoming an important option in breast cancer treatment. In triple-negative breast cancer (TNBC), pembrolizumab showed potential benefits in high-risk early-stage disease and metastases (1,2). In estrogen receptor-positive, HER2-negative (ER+/HER2-) early-stage breast cancer, tumors with lower ER and higher PD-1 were more likely to respond to neoadjuvant immune checkpoint inhibitors (ICI) (3,4). However, most trials excluded invasive lobular breast cancer, and the investigation of clinical biomarkers beyond ER, PD-1, or TILs is limited.

Transcriptomic data is useful to identify prognostic biomarkers, including tumor subtypes and immune patterns. The intrinsic molecular subtype was one of the first classification systems derived from patient tumor RNA expression (5). In TCGA, three ILC subtypes were revealed from tumor RNA-seq data by unsupervised clustering, where the reactive-like subtype had better disease free survival and overall survival than the proliferative subtype (6). Similar classifications were also derived in the RATHER study (7). More recently, four transcriptomic subtypes were defined in HR+/HER2- breast cancer, among which the receptor tyrosine kinase (RTK)-driven subtype had poorest outcomes compared to the canonical luminal, immunogenic, and proliferation subtypes (8). For immune patterns, immune quantification data is scarce, therefore, to relate to clinical prognosis, studies often calculated scores from marker gene expression of individual immune cell types in bulk RNA-seq data. For example, one study reported that higher immune infiltration was linked with lower tumor purity, and predicted better overall survival (9). Another study analyzed TCGA and METABRIC data, and found that tumors with higher M1-like signature or M1/M2 ratio were likely more aggressive with poorer survival (10). However, prognostic impact of immune infiltration patterns remains ambiguous, considering decreased estimation accuracy for more complex immune cell subtype composition. Furthermore, most studies excluded ILCs, which had distinct immune infiltration than no special type (NST) tumors (11,12), thus introducing bias especially in data-driven conclusions. Further research is thus warranted to identify prognostic immune infiltration patterns in ILC tumors specifically.

In this study, we performed integrative analysis of both RNA-seq and multispectral immunohistochemistry (mIHC) data in primary ER+/HER2- ILC. We identified two transcriptomic ILC subtypes, five immune infiltration patterns, and linked the ILC subtypes with immune infiltration. We then correlated gene expression with immune cell density, and defined a tumor associated macrophage (TAM)-Low signature, which was negatively associated with TAM infiltration. We further showed in public datasets that TAM-Low was inversely correlated with presence of proliferating TAMs, and predicted improved survival outcomes in ER+ breast cancer.

21 primary ER+ treatment-naïve lobular breast tumors were included in our cohort (Supplementary Table 1). We performed RNA-seq in all samples, and mIHC in 13 of the tumors using the same FFPE block (Fig. 1A). We identified *CDH1* truncating mutations in 10 samples from RNA-seq (Supplementary Table 2, Supplementary Figure 1A). We then computed transcriptomic subtypes. Using TCGA classifier (6), we identified 12 proliferative, three reactive-like, and six immune-related tumors, and combined the latter two groups as the non-proliferative subtype (Fig. 1B). Consistently, we found higher expression of cell-cycle pathways in the proliferative group, and higher expression of immune-related pathways in the non-proliferative subtype, using reference pathways from the RATHER ILC cohort (enriched pathways of hormone-related and immune-related subtypes, respectively) (Fig. 1C). Specifically, the non-proliferative subtype had higher stromal score and mostly consisted of luminal A and normal-like subtypes. In contrast, the proliferative subtype had higher tumor cell purity, lower stromal composition, and was composed of multiple PAM50 types, including luminal A, luminal B, basal, and HER2-enriched subtypes (Fig. 1D-E).

Next, we examined mIHC immune infiltration patterns from ROIs in tumor and stromal regions (Fig. 2A). In general, macrophage showed highest infiltration, followed by CD4 T cells and CD8 T cells, all with higher infiltration in stromal than tumor regions (Fig. 2B). Correlation based on infiltration density of the different immune cells, reflecting their associations within or between tumor and stroma, we observed three clusters - stromal and tumor B cells, Treg cells and macrophages, and CD4 T cells and CD8 T cells (Fig. 2C). Furthermore, we identified five immune infiltration patterns in both stromal and tumor regions from non-negative matrix factorization (NMF) clustering (Fig. 2D). In stromal regions, pattern 1, 3, and 5 had generally low infiltration of all immune cells, pattern 2 had higher macrophages and Tregs, and pattern 4 had higher infiltration of CD4 T cells and CD8 T cells (Fig. 2E-F). Similar immune infiltrating patterns were observed in tumor regions (Supplementary Figure 1D-E). This suggested a potential immunosuppressive microenvironment in pattern 2 while more immune active environment in patterns 4. Specifically, within each patient, the tumors had up to 5 immune patterns, and was often dominated by 1 (blue arrows) or 2 (orange arrows) co-existing patterns (Supplementary Figure 1B).

We then linked immune infiltration with the ILC transcriptomic subtypes, defined in the previous section. The proliferative subtype showed higher infiltration of almost all immune cell types at both stromal and tumor areas than the non-proliferative subtype (Fig. 2G-H). Specifically, pattern 2 (likely immunosuppressive) predominated in the proliferative subtype (Supplementary Figure 1C). Altogether, it suggested higher immune infiltration, and potentially more immunosuppressive cell types in the proliferative than non-proliferative ILCs.

Access to both mIHC as well as mRNA expression data allowed us to analyze correlations between gene expression and immune cell infiltration. We therefore calculated Spearman's correlation between expression of each individual gene (26,485 genes, TPM) and infiltration of major immune cell type (5 types) in stromal or tumor regions (median number per $mm^2$ among ROIs), including all possible pairwise combinations. Filtering by FDR<0.05, we identified 651 significant correlations between expression of genes and immune cell subtypes (Fig. 3A). The majority of correlations were negative associations between gene expression and macrophage infiltration. Combining genes inversely related with presence of stromal or tumor macrophages, we generated a 'TAM-Low' signature of 483 genes (Fig. 3A, Supplementary Figure 2A), which was enriched in extracellular matrix pathways (Fig. 3B).

Next we compared signature expression of TAM-Low with marker genes of multiple subtypes of tumor-associated macrophages (Supplementary Table 3) from literature in ER+ primary breast cancer, using RNA-seq of METABRIC and SCAN-B (13-15). In both datasets, the TAM-Low signature was negatively correlated with proliferating TAMs (Prolif-TAMs) (Fig 3C), a pro-inflammatory subtype characterized by high expression of HMGB1 and cell cycle-related genes (CCNA2, CDC45, CDK1, MKI67, RRM2, STMN1, TOP2A, and TYMS). In summary, we identified a negative association between the TAM-low signature and tumor associated macrophages, and specifically our data suggested that TAM-low genes such as those from the extracellular matrix (ECM) pathway might hinder the infiltration of proliferating, pro-inflammatory tumor associated macrophages.

And finally, we investigated association of TAM-Low with both survival and therapy response. In ER+ breast cancer, the TAM-Low signature predicted improved relapse free survival as well as overall survival in METABRIC ER+ tumors, adjusting for multiple demographic and clinical variables in multivariate Cox regression (Fig. 3D-E). Result in SCAN-B ER+ tumors was similar but lacked significance (Supplementary Figure 2C). For therapy response, we analyzed the TAM-Low signature levels in ER+/HER2- primary breast tumors from patients treated with neoadjuvant aromatase inhibitor (AI) in the POETIC trial (16). Tumors from AI responders had higher median value TAM-Low signature expression than non-responders (Fig. 3F-H) at baseline and more pronounced after neoadjuvant retreatment, although this did not reach significance. In line with our prior observation of a negative association between the TAM-Low signature and marker genes of proliferating TAMs (Prolif-TAMs), we observed significantly lower proliferating TAMs (Prolif-TAM) after AI treatment in the responders. Using expression of marker genes of other TAM subtypes (15), we failed to identify another set of TAMs significantly different between non-responders and responders, except for lower interferon-primed TAMs (IFN-TAM) at baseline in responders (Fig. 3F). In summary, this data suggests that the TAM-low signature is associated with improved endocrine therapy response and outcome ER+ breast cancer.

Finding biomarkers is essential to guide precision immune therapy in breast cancer. In TNBC, higher tumor-infiltrating lymphocytes and mutation burden predicted higher ICI response rate. In early HR+/HER2- cancer, which had relatively lower mutation incidence and immune infiltration, positive lymph node, ER%<10%, and combined positive score ≥1 were associated with higher pCR rate with neoadjuvant pembrolizumab treatment (3), and elevated PD-L1 scores, ER%<50%, PR%<10%, and stromal TILs ≥1% indicated pCR and no residual cancer burden with neoadjuvant nivolumab therapy (4). However, clinical associations of other immune cell types or genes were understudied, and whether pCR translates to long-term survival benefit remained unclear. Moreover, clinical trials did not focus on lobular breast cancer, which had a somewhat distinct immune landscape than NSTs (11,17,18).

Our study focused on primary ER+/HER2- early-stage ILC, and found ILC transcriptomic subtypes were closely related with immune infiltration phenotypes. Specifically, in the proliferative ILC subtype greater infiltration of multiple immune cell types, higher occurrence of immuno-suppressive patterns was also observed. This was consistent with clinical observations that high TIL level in ILC was associated with young age, proliferative tumors, and lymph node invasion(18). From our previous study, higher macrophage infiltration was related to lower recurrence rate in IDC, but this was not observed in ILC (11). In general, large-scale data is lacking for clinical outcome associations with TIL subtypes or other immune cell types. This hence warrants future effort to incorporate mIHC quantification in prospective cohorts, and to include ILC cases in clinical trials (17).

Most current immunotherapies focus on lymphocytes. However, macrophages, rather than T cells, were dominant in ILCs (11). In our study, we also found macrophage infiltration as the 'hub' in association with tumor gene expression. Our TAM-Low signature which was directly derived from correlation analysis between gene expression and immune infiltration based on mIHC has potential as a promising biomarker, predicting low infiltration of proinflammatory Prolif-TAMs, and improved survival in patients with ER+ breast cancer. Furthermore, higher Prolif-TAMs likely marks intrinsic aromatase inhibitor resistance in ER+/HER2- primary breast tumors. As resident tumor macrophages expressing MKI67, CDK1, and CDC45, HMGB1, and others, Prolif-TAMs were likely pro-inflammatory, profibrotic, and promoting tumor progression (15). In pancreatic ductal adenocarcinoma, targeting Prolif-TAM promoted anti-tumor response via cytotoxic CD8+ T cell redistribution (19). Interestingly, from our original IDC and ILC cohort, we had reported a subgroup of cycling monocytes/macrophages (cluster 4) expressing MKI67 and SPP1 genes, which likely corresponded to Prolif-TAMs (11). Of note, each TAM subtype affects breast cancer differently. For example, the FOLR2+ tumor-stroma-resident macrophages correlated to higher T cell infiltration and better prognosis (20). Conversely, TREM2+ macrophages likely determined an immunosuppressive metastatic niche, with TREM2 predicting poorer overall survival and relapse-free survival in triple-negative breast cancer (21,22). Therefore, we encourage quantification of individual TAM subtypes in breast cancer and specifically in ILC as a more accurate immune phenotyping in clinical response prediction.

Limitations of this study include small cohort size, variations in ROI selection, and absence of immunophenotyping panel of a broader range of immune subtypes, including the macrophage population. There are also limitations to combining different methodologies for analysis of immune cell infiltration and activities, as reflected by some discrepancy between mIHC and RNA-seq deconvolution algorithms – e.g., proliferative ILCs had higher stromal or immune scores and showed lower expression of immune signaling than non-proliferative ones, including the JAK-STAT pathway and local acute inflammatory response (LAIR pathway). A possible explanation is that immune signals were expressed by tumor cells, thus leading to spillover effect and elevated immune scores in non-proliferative tumor (23).

In conclusion, through integrated RNA-seq and mIHC analysis of ER+/HER2- primary ILC tumors, we found that the proliferative ILC transcriptomic subtype exhibited higher immune infiltration. We would like to propose the TAM-Low signature as a potential novel biomarker for predicting reduced infiltration of pro-inflammatory and pro-tumorigenic macrophages and improved survival in ER+ breast cancer, and encourage its further analysis in additional studies. Future studies are warranted to quantify TAM subtypes in ILC, as indicators of endocrine treatment response and survival, as well as to improve potential use of immunotherapy.

## Methods

### Study design, sample acquisition, and sample processing

Patient recruitment was described previously (11). For the 21 cases, formalin-fixed paraffin-embedded (FFPE) blocks of primary ER+/HER2- ILC tumors were acquired from University of Pittsburgh Biospecimen Core, and clinical information was queried from the UPMC Cancer Registry. For each block, three sections (at the top, bottom and middle of the block) were stained with hematoxylin and eosin (H&E) and inspected by pathologist to circle out regions with tumor epithelial cell proportion ≥40%. 5 sections (of 10uM thickness each) were used for RNA extraction and sequencing at these circled regions. And another 3 sections (of 4uM thickness each) were used for multispectral immunophenotyping by mIHC. Protocol of this study was approved by Institutional Review Boards at University of Pittsburgh (protocol numbers PRO17100602 and PRO16060685).

### RNA sequencing and bioinformatics analysis

Sample RNA was extracted with Qiagen tissue DNA/RNA kit from FFPE sections, assessed with High Sensitivity RNA TapeStation for quality and concentration, and sequenced with Illumina Nextseq 500 75 bp pair-end in 150 cycles at University of Pittsburgh Health Sciences Sequencing Core. For RNA-seq data, we used FastQC for FASTQ data quality control, trimmomatic for adapter trimming, Hisat2 for alignment to human genome 38 (hg38), and HTSeq for quantification, to generate normalized expression in transcripts per million (TPM). To call CDH1 mutations from RNA-seq data, we used GATK v4 MuTect2 pipeline (broadinstitute.org) to call all somatic SNVs and indels from bam files calculated with STAR (v2.7.5a), and selected CDH1 mutations from annotation from ANNOVAR (24). For ILC subtyping, we first trained a Nearest Centroid classifier in original TCGA ILC samples with RNA expression of the 60-gene signature, using original annotation of the proliferative, reactive-like, and immune-related subtypes (6). We then applied the trained classifier to RNA-seq data of this study, which generated one subtype for each sample. Both training and testing sets were normalized in $log_2$TPM and transformed into gene-wise z-score. Finally, we combined the reactive-like and immune-related subgroups as the 'non-proliferative' subtype, due to limited sample numbers of both classes. We used GSVA (v1.48.2, ssGSEA) to calculate signature scores of immune-related and cell-cycle related pathways from TPM of our samples. We calculated PAM50 subtype probability with Genefu (v2.26.0), and estimated tumor purity, stromal score, and immune scores using deconvolute_estimate (immunedeconv v2.1.0).

### mIHC staining, quantification, and statistical analysis

Workflow of mIHC was described with processed data (median infiltration of each immune cell type at patient level) previously (11). In brief, FFPE sections of 4uM thickness were stained with an immunophenotyping panel (CD20, CD4, CD8, FOXP3, CD68, Pan-CK), from which immune cells were counted using InForm software from randomly selected regions of interests (ROI) (3-13 / section) at representative immune infiltration areas, from Vectra Polaris (PerkinElmer) scans at x10. Specifically, infiltration of each immune cell type was calculated in tumor and stromal regions respectively (number/$mm^2$). We computed Spearman's correlation for abundance of each pair of immune cell types (number/$mm^2$) and used FDR<0.05 to select statistically significant correlations. Non-negative matrix factorization was used to cluster ROIs based on Spearman's correlation matrix of $log_2$ normalized counts (BigNmf, v1.0.0). from which 5 clusters were selected based on elbow point of cophenetic correlation coefficient. The corresponding consensus matrix was plotted in Euclidean distance in Ward's linkage.

### TAM-Low signature generation and pathway analysis

We calculated Spearman's correlation between expression of each gene (TPM) and abundance of each immune cell type (number/$mm^2$) at tumor and stromal region (median values among ROIs). A gene-immune cell pair correlation is significant if with FDR<0.05. Genes which were significantly negatively correlated with stromal or tumor macrophage infiltration were included into 'TAM-Low' signature (483 genes, Fig. 3, Supplementary Figure 2). Pathway analysis was performed with EnrichR, using Gene Ontology, Kyoto Encyclopedia of Genes and Genomes (KEGG), and Reactome datasets (25).

#### Public datasets and analysis

Signatures of TAM subtypes were downloaded (15). We used consensus TME algorithm (immunedeconv v2.1.0, deconvolute_consensus_tme_custom) (23,26) to calculate deconvolution score for TAM-Low and TAM subtype signatures from normalized expression of samples in METABRIC (normalized expression, downloaded from cBioportal) (14), SCAN-B ($log_2$ counts per million)(13), and POETIC trial (27). Spearman's correlation was calculated between signature scores of every pair of TAM subtypes among samples with FDR correction. In survival analysis, we classified samples by 'TAM-Low' signature expression (high vs low) using optimal cut point (survminer v0.4.9), and calculated hazard ratio from multivariate Cox regression, adjusting for histology, HER2, PR, age at diagnosis, tumor size, and tumor stage (METABRIC (14) and SCAN-B (13)). For POETIC trial data, RNA-seq and responder information were downloaded from GSE105777 and GSE126870 (16).

### Data availability

The raw and processed data will be available in GEO upon publication.

### Code availability

Codes for data processing will be available on github upon publication, or via reasonable request from the corresponding author.

### Acknowledgements

This work was supported by the National Institutes of Health (R35 CA263850, and P01 AI108545 (D.A.A.V.)), NIH R01 CA252378 (S. Oesterreich and A.V.L.)), BCRF (S. Oesterreich and A.V.L.), The Shear Family Foundation and Magee Womens Research Institute and Foundation. Pittsburgh Genomics Core and Center for Research Computing (RRID: SCR_022735) for computational resources and RNA-seq services was supported by NIH S10OD028483. The funders had no role in study design, data collection and analysis, decision to publish or preparation of this paper.

### Author contributions

SO (Steffi Oesterreich) and SO (Sayali Onkar) developed concept and design of this study. SO (Sayali Onkar), YL, and PL accomplished data acquisition and pre-processing. FC performed formal data analysis, data interpretation, statistical analysis, and drafted the manuscript. JZ and YL provided administrative, technical, or material support in data analysis. GT, TB, DAAV, SO (Steffi Oesterreich), and AVL provided supervision. All authors contributed to critical revision of the manuscript for important intellectual content.

### Competing interests

DAAV: cofounder and stock holder – Novasenta, Potenza, Tizona, Trishula; stock holder – Werewolf; patents licensed and royalties - BMS, Novasenta; scientific advisory board member - Werewolf, F-Star, Apeximmune, T7/Imreg Bio; consultant - BMS, Regeneron, Ono Pharma, Avidity Partners, Peptone; funding - BMS, Novasenta. Other authors declare no competing interests.

## References

1. Cortes J, Rugo HS, Cescon DW, Im S-A, Yusof MM, Gallardo C*, et al.* Pembrolizumab plus chemotherapy in advanced triple-negative breast cancer. New England Journal of Medicine **2022**;387(3):217-26.
2. Schmid P, Cortes J, Pusztai L, McArthur H, Kümmel S, Bergh J*, et al.* Pembrolizumab for early triple-negative breast cancer. New England Journal of Medicine **2020**;382(9):810-21.
3. Cardoso F, Bardia A, Andre F, Cescon DW, McArthur HL, Telli ML*, et al.* KEYNOTE-756: Randomized, double-blind, phase 3 study of pembrolizumab vs placebo combined with neoadjuvant chemotherapy and adjuvant endocrine therapy for high-risk, early-stage estrogen receptor–positive, human epidermal growth factor receptor 2–negative (ER+/HER2−) breast cancer. American Society of Clinical Oncology; 2019.
4. Loi S, McArthur HL, Harbeck N, Pusztai L, Delaloge S, Letrent K*, et al.* A phase III trial of nivolumab with neoadjuvant chemotherapy and adjuvant endocrine therapy in ER+/HER2- primary breast cancer: CheckMate 7FL. American Society of Clinical Oncology; 2020.
5. Perou CM, Sørlie T, Eisen MB, Van De Rijn M, Jeffrey SS, Rees CA*, et al.* Molecular portraits of human breast tumours. nature **2000**;406(6797):747-52.
6. Ciriello G, Gatza ML, Beck AH, Wilkerson MD, Rhie SK, Pastore A*, et al.* Comprehensive molecular portraits of invasive lobular breast cancer. Cell **2015**;163(2):506-19.
7. Michaut M, Chin S-F, Majewski I, Severson TM, Bismeijer T, De Koning L*, et al.* Integration of genomic, transcriptomic and proteomic data identifies two biologically distinct subtypes of invasive lobular breast cancer. Scientific reports **2016**;6(1):1-13.
8. Jin X, Zhou Y-F, Ma D, Zhao S, Lin C-J, Xiao Y*, et al.* Molecular classification of hormone receptor-positive HER2-negative breast cancer. Nature Genetics **2023**;55(10):1696-708.
9. Xu Q, Yan X, Han Z, Jin X, Jin Y, Sun H*, et al.* Immune cell infiltration and relevant gene signatures in the tumor microenvironment that significantly associates with the prognosis of patients with breast cancer. Frontiers in Molecular Biosciences **2022**;9:823911.
10. Oshi M, Tokumaru Y, Asaoka M, Yan L, Satyananda V, Matsuyama R*, et al.* M1 Macrophage and M1/M2 ratio defined by transcriptomic signatures resemble only part of their conventional clinical characteristics in breast cancer. Scientific reports **2020**;10(1):16554.
11. Onkar S, Cui J, Zou J, Cardello C, Cillo AR, Uddin MR*, et al.* Immune landscape in invasive ductal and lobular breast cancer reveals a divergent macrophage-driven microenvironment. Nature Cancer **2023**;4(4):516-34.
12. Du T, Zhu L, Levine KM, Tasdemir N, Lee AV, Vignali DA*, et al.* Invasive lobular and ductal breast carcinoma differ in immune response, protein translation efficiency and metabolism. Scientific reports **2018**;8(1):1-11.
13. Saal LH, Vallon-Christersson J, Häkkinen J, Hegardt C, Grabau D, Winter C*, et al.* The Sweden Cancerome Analysis Network-Breast (SCAN-B) Initiative: a large-scale multicenter infrastructure towards implementation of breast cancer genomic analyses in the clinical routine. Genome medicine **2015**;7:1-12.
14. Curtis C, Shah SP, Chin S-F, Turashvili G, Rueda OM, Dunning MJ*, et al.* The genomic and transcriptomic architecture of 2,000 breast tumours reveals novel subgroups. Nature **2012**;486(7403):346-52.
15. Ma R-Y, Black A, Qian B-Z. Macrophage diversity in cancer revisited in the era of single-cell omics. Trends in immunology **2022**;43(7):546-63.
16. Gao Q, López-Knowles E, Cheang MCU, Morden J, Ribas R, Sidhu K*, et al.* Impact of aromatase inhibitor treatment on global gene expression and its association with antiproliferative response in ER+ breast cancer in postmenopausal patients. Breast Cancer Research **2020**;22:1-20.
17. Van Baelen K, Van Cauwenberge J, Maetens M, Beck G, Camden A, Chase M-C*, et al.* Reporting on invasive lobular breast cancer in clinical trials: a systematic review. NPJ Breast Cancer **2024**;10(1):23.

18. Desmedt C, Salgado R, Fornili M, Pruneri G, Van den Eynden G, Zoppoli G*, et al.* Immune infiltration in invasive lobular breast cancer. JNCI: Journal of the National Cancer Institute **2018**;110(7):768-76.
19. Yang X, Lin J, Wang G, Xu D. Targeting proliferating tumor-infiltrating macrophages facilitates spatial redistribution of CD8+ T cells in pancreatic cancer. Cancers **2022**;14(6):1474.
20. Ramos RN, Missolo-Koussou Y, Gerber-Ferder Y, Bromley CP, Bugatti M, Núñez NG*, et al.* Tissue-resident FOLR2+ macrophages associate with CD8+ T cell infiltration in human breast cancer. Cell **2022**;185(7):1189-207. e25.
21. Molgora M, Esaulova E, Vermi W, Hou J, Chen Y, Luo J*, et al.* TREM2 modulation remodels the tumor myeloid landscape enhancing anti-PD-1 immunotherapy. Cell **2020**;182(4):886-900. e17.
22. Yofe I, Shami T, Cohen N, Landsberger T, Sheban F, Stoler-Barak L*, et al.* Spatial and temporal mapping of breast cancer lung metastases identify TREM2 macrophages as regulators of the metastatic boundary. Cancer discovery **2023**;13(12):2610-31.
23. Sturm G, Finotello F, Petitprez F, Zhang JD, Baumbach J, Fridman WH*, et al.* Comprehensive evaluation of transcriptome-based cell-type quantification methods for immuno-oncology. Bioinformatics **2019**;35(14):i436-i45.
24. Wang K, Li M, Hakonarson H. ANNOVAR: functional annotation of genetic variants from high-throughput sequencing data. Nucleic acids research **2010**;38(16):e164-e.
25. Kuleshov MV, Jones MR, Rouillard AD, Fernandez NF, Duan Q, Wang Z*, et al.* Enrichr: a comprehensive gene set enrichment analysis web server 2016 update. Nucleic acids research **2016**;44(W1):W90-W7.
26. Jiménez-Sánchez A, Cast O, Miller ML. Comprehensive benchmarking and integration of tumor microenvironment cell estimation methods. Cancer Research **2019**;79(24):6238-46.
27. Anders S, Pyl PT, Huber W. HTSeq—a Python framework to work with high-throughput sequencing data. bioinformatics **2015**;31(2):166-9.

## Figure Legends

**Figure 1. Study design and ILC subtype identification from RNA-seq**

A. Study design and workflow. Treatment-naïve ER+/HER2- primary ILC tumors were processed as FFPE blocks, from which FFPE sections underwent RNA-seq in tumor-rich regions selected by pathologist (N=21), and multispectral IHC (mIHC) with immunophenotyping panel (N=13). For mIHC, immune cell infiltration was quantified in regions of interest (ROIs) in both tumor and stromal area. B. Expression of ILC classifier genes in the ER+/HER2- ILC cohort, showing the two ILC subtypes (proliferative in orange box, non-proliferative (immune + reactive) in blue box), PAM50 subtypes, and other demographic and clinical information. C. Signature score (ssGSEA score) of reference ILC subtype pathways from RATHER study. Only pathways with significant signature score difference between non-proliferative and proliferative ILC subtypes were shown (Mann-Whitney-Wilcoxon test). D. Estimated tumor purity, stromal score, and immune score from PUREE and ESTIMATE algorithm. E. PAM50 subtype composition of non-proliferative and proliferative ILC tumors.

**Figure 2. Immune cell infiltration landscape from mIHC**

A. Immune cell infiltration in tumor and stromal regions among ROIs (number/mm$^2$), annotated with ILC cluster, PAM50 subtypes, and demographic or clinical information. B. Histogram of immune cell type abundance (/mm$^2$) in stromal and tumor region ROIs. C. Spearman correlation (FDR<0.05) of immune cell infiltration (number/mm$^2$) between cell types. D. NMF consensus matrix from immune cell infiltration in both stromal and tumor regions among ROIs, showing 5 immune infiltration patterns. E. Stromal region immune cell infiltration (number/mm$^2$) among immune infiltration patterns, showing each pattern per panel. F. Stromal region immune cell infiltration (number/mm$^2$) among immune infiltration patterns, showing each immune cell type per panel. ****p<0.0001, ***p<0.001, **p<0.01, *p<0.05, comparison (horizontal brackets) with no label, p≥0.05. G-H. Immune cell infiltration (number/mm$^2$) in proliferative and non-proliferative ILC tumors in stromal (G) and tumor regions (H).

**Figure 3. Derivation and clinical associations of TAM-Low signature**

A. Number of gene-cell pairs with significant (FDR<0.05) positive or negative Spearman's correlation between gene expression (TPM) and immune cell infiltration (median value among ROIs in number/mm2). B. Pathway enrichment of TAM-Low signature genes (483 genes). C. Spearman correlation (FDR<0.05) between TAM-Low and other TAM subtype signatures in

METABRIC ER+ tumors (N=1,505). D-E. Hazard ratio of relapse free survival (D) and overall survival (E) of TMA-Neg signature (deconvolution score) and demographic-clinical variables in METABRIC ER+ tumors (N=1,096, excluding entries with missing data in any of the regression covariates) from multivariant Cox regression. F-H. Deconvolution score of TAM-Low signature and TAM subtype signatures in baseline (F), post-aromatase-inhibitor (AI) treatment tumors (G), and change after treatment (Δ(Post-AI – baseline)) (H) from POETIC trial cohort of 131 patients. IFN-TAMs, interferon-primed TAMs; Reg-TAMs, immune regulatory TAMs, Inflam-TAMs, inflammatory cytokine-enriched TAMs; Angio-TAMs, proangiogenic TAMs; LA-TAMs, lipid-associated TAMs; Prolif-TAMs, proliferating TAMs.

## Figure 1

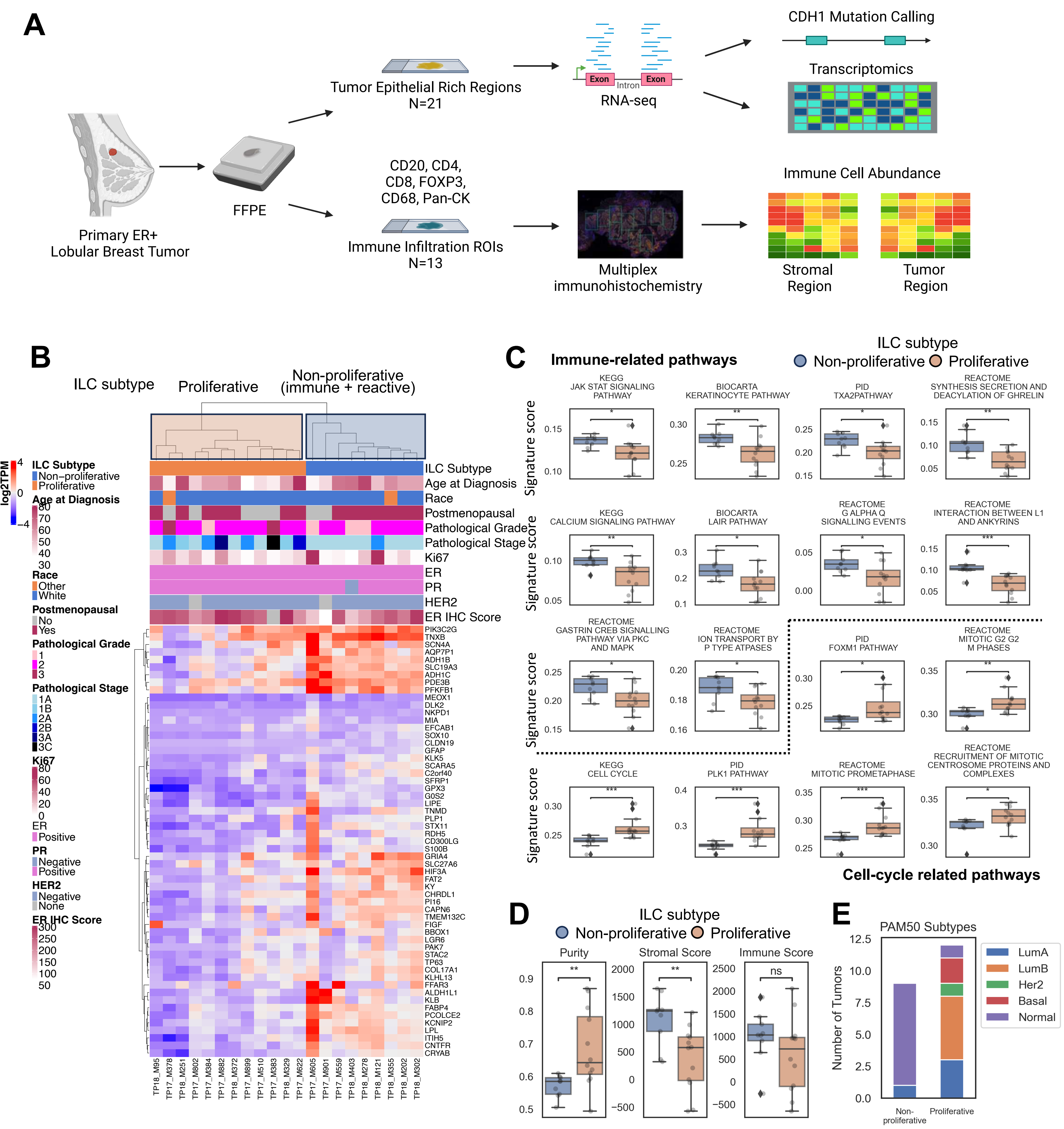

A
Primary ER+ Lobular Breast Tumor
FFPE
Tumor Epithelial Rich Regions N=21
CD20, CD4, CD8, FOXP3, CD68, Pan-CK
Immune Infiltration ROIs N=13
Exon
Intron
RNA-seq
Multiplex immunohistochemistry
CDH1 Mutation Calling
Transcriptomics
Immune Cell Abundance
Stromal Region
Tumor Region
B
ILC subtype
Proliferative
Non-proliferative (immune + reactive)
ILC Subtype
Age at Diagnosis
Race
Postmenopausal
Pathological Grade
Pathological Stage
Ki67
ER
PR
HER2
ER IHC Score
C
Immune-related pathways
Cell-cycle related pathways
Non-proliferative
Proliferative
Signature score
D
Purity
Stromal Score
Immune Score
E
PAM50 Subtypes
Number of Tumors
LumA
LumB
Her2
Basal
Normal

## Figure 2

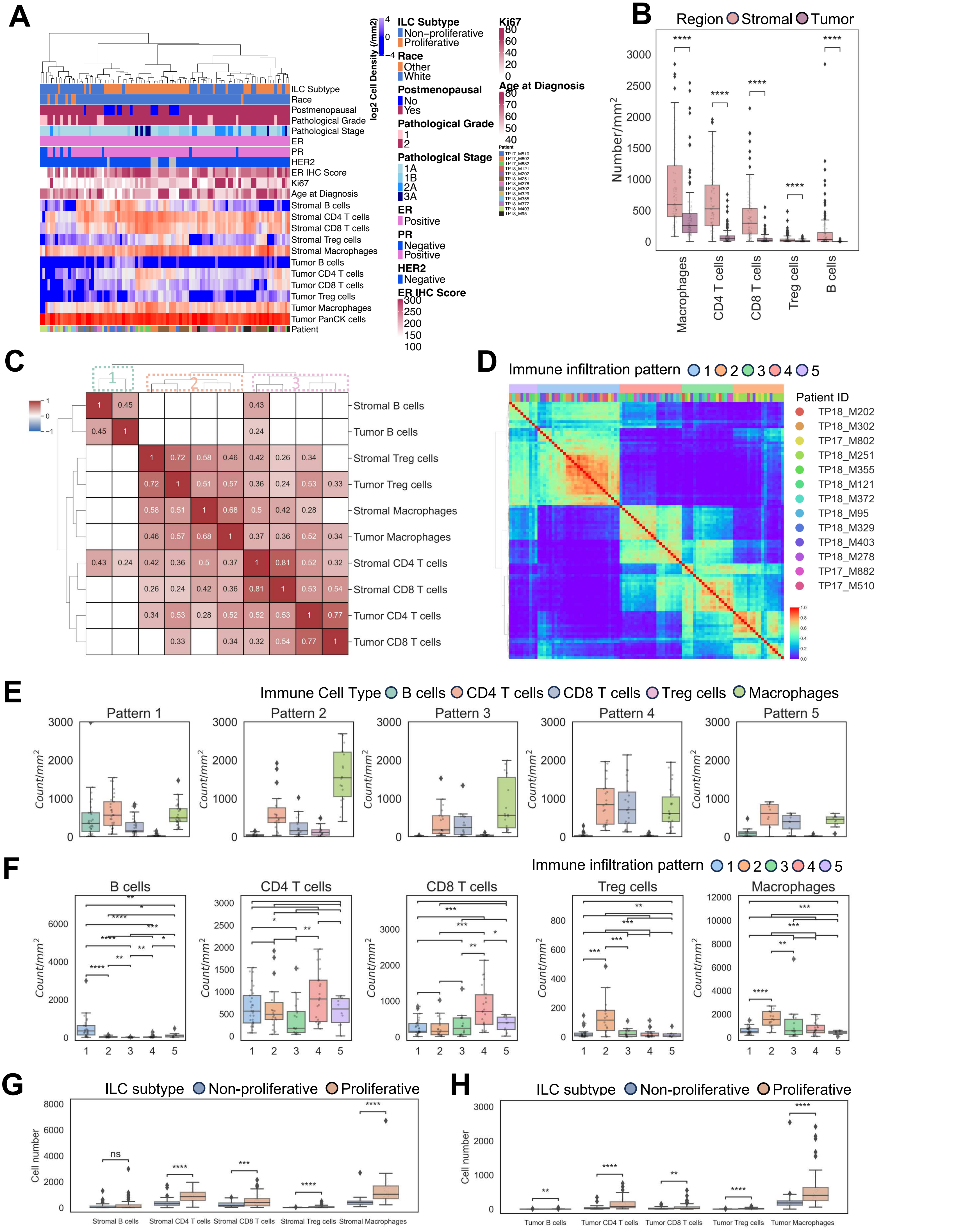

A
ILC Subtype
Race
Postmenopausal
Pathological Grade
Pathological Stage
ER
PR
HER2
ER IHC Score
Ki67
Age at Diagnosis
Stromal B cells
Stromal CD4 T cells
Stromal CD8 T cells
Stromal Treg cells
Stromal Macrophages
Tumor B cells
Tumor CD4 T cells
Tumor CD8 T cells
Tumor Treg cells
Tumor Macrophages
Tumor PanCK cells
Patient
log2 Cell Density (/mm2)
ILC Subtype
Non−proliferative
Proliferative
Race
Other
White
Postmenopausal
No
Yes
Pathological Grade
1
2
Pathological Stage
1A
1B
2A
3A
ER
Positive
PR
Negative
Positive
HER2
Negative
ER IHC Score
Ki67
Age at Diagnosis
B
Region
Stromal
Tumor
Number/mm2
Macrophages
CD4 T cells
CD8 T cells
Treg cells
B cells
C
Stromal B cells
Tumor B cells
Stromal Treg cells
Tumor Treg cells
Stromal Macrophages
Tumor Macrophages
Stromal CD4 T cells
Stromal CD8 T cells
Tumor CD4 T cells
Tumor CD8 T cells
D
Immune infiltration pattern 1 2 3 4 5
Patient ID
TP18_M202
TP18_M302
TP17_M802
TP18_M251
TP18_M355
TP18_M121
TP18_M372
TP18_M95
TP18_M329
TP18_M403
TP18_M278
TP17_M882
TP17_M510
E
Immune Cell Type
B cells
CD4 T cells
CD8 T cells
Treg cells
Macrophages
Pattern 1
Pattern 2
Pattern 3
Pattern 4
Pattern 5
Count/mm2
F
Immune infiltration pattern 1 2 3 4 5
B cells
CD4 T cells
CD8 T cells
Treg cells
Macrophages
Count/mm2
G
ILC subtype
Non-proliferative
Proliferative
Cell number
Stromal B cells
Stromal CD4 T cells
Stromal CD8 T cells
Stromal Treg cells
Stromal Macrophages
H
ILC subtype
Non-proliferative
Proliferative
Cell number
Tumor B cells
Tumor CD4 T cells
Tumor CD8 T cells
Tumor Treg cells
Tumor Macrophages

## Figure 3

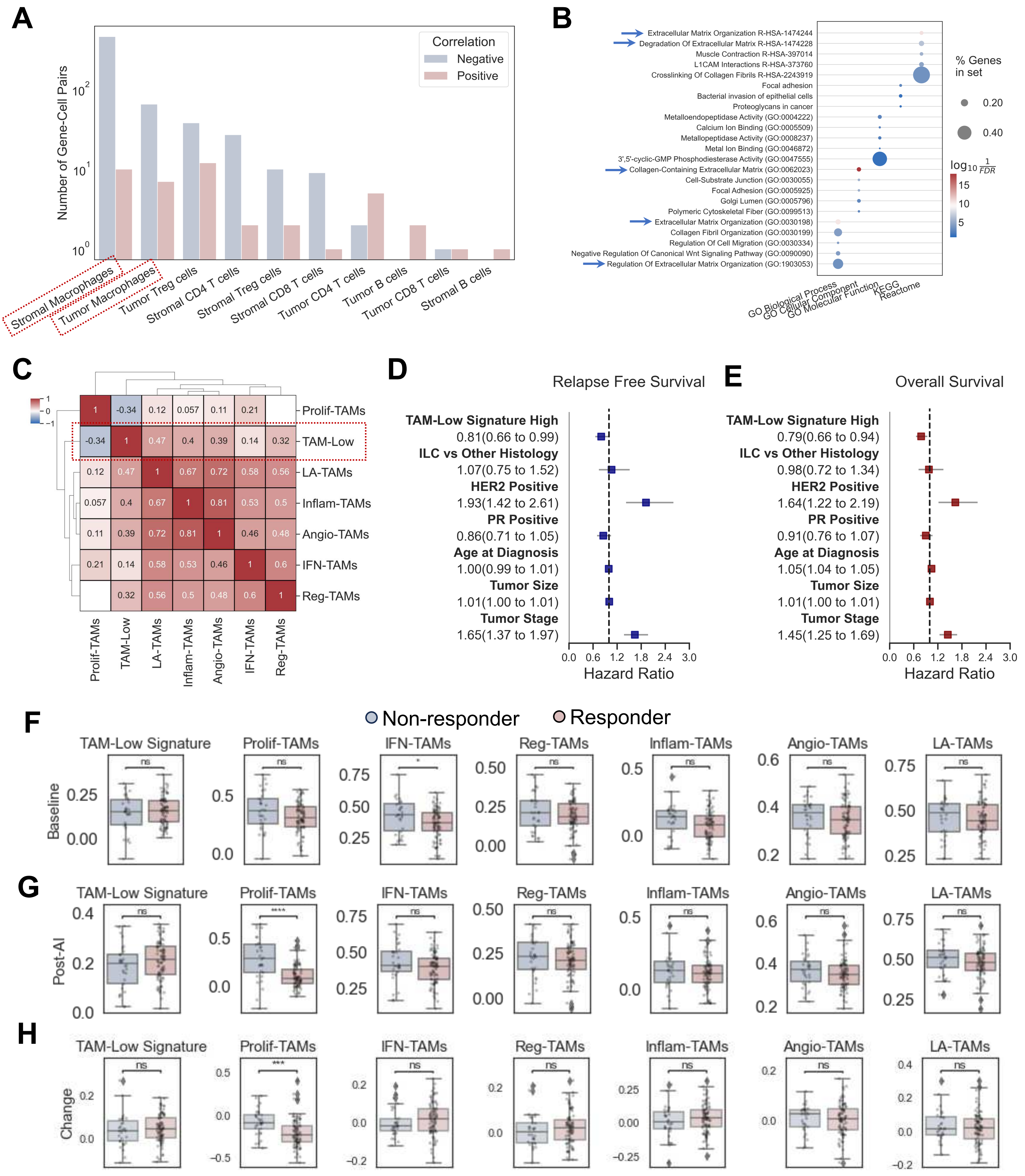

A
Correlation
Negative
Positive
Number of Gene-Cell Pairs
Stromal Macrophages
Tumor Macrophages
Tumor Treg cells
Stromal CD4 T cells
Stromal Treg cells
Stromal CD8 T cells
Tumor CD4 T cells
Tumor B cells
Tumor CD8 T cells
Stromal B cells
B
Extracellular Matrix Organization R-HSA-1474244
Degradation Of Extracellular Matrix R-HSA-1474228
Muscle Contraction R-HSA-397014
L1CAM Interactions R-HSA-373760
Crosslinking Of Collagen Fibrils R-HSA-2243919
Focal adhesion
Bacterial invasion of epithelial cells
Proteoglycans in cancer
Metalloendopeptidase Activity (GO:0004222)
Calcium Ion Binding (GO:0005509)
Metallopeptidase Activity (GO:0008237)
Metal Ion Binding (GO:0046872)
3',5'-cyclic-GMP Phosphodiesterase Activity (GO:0047555)
Collagen-Containing Extracellular Matrix (GO:0062023)
Cell-Substrate Junction (GO:0030055)
Focal Adhesion (GO:0005925)
Golgi Lumen (GO:0005796)
Polymeric Cytoskeletal Fiber (GO:0099513)
Extracellular Matrix Organization (GO:0030198)
Collagen Fibril Organization (GO:0030199)
Regulation Of Cell Migration (GO:0030334)
Negative Regulation Of Canonical Wnt Signaling Pathway (GO:0090090)
Regulation Of Extracellular Matrix Organization (GO:1903053)
GO Biological Process
GO Cellular Component
GO Molecular Function
KEGG
Reactome
% Genes in set
0.20
0.40
log10 1/FDR
15
10
5
C
Prolif-TAMs
TAM-Low
LA-TAMs
Inflam-TAMs
Angio-TAMs
IFN-TAMs
Reg-TAMs
D
Relapse Free Survival
TAM-Low Signature High
0.81(0.66 to 0.99)
ILC vs Other Histology
1.07(0.75 to 1.52)
HER2 Positive
1.93(1.42 to 2.61)
PR Positive
0.86(0.71 to 1.05)
Age at Diagnosis
1.00(0.99 to 1.01)
Tumor Size
1.01(1.00 to 1.01)
Tumor Stage
1.65(1.37 to 1.97)
Hazard Ratio
E
Overall Survival
TAM-Low Signature High
0.79(0.66 to 0.94)
ILC vs Other Histology
0.98(0.72 to 1.34)
HER2 Positive
1.64(1.22 to 2.19)
PR Positive
0.91(0.76 to 1.07)
Age at Diagnosis
1.05(1.04 to 1.05)
Tumor Size
1.01(1.00 to 1.01)
Tumor Stage
1.45(1.25 to 1.69)
Hazard Ratio
F
Non-responder
Responder
TAM-Low Signature
Prolif-TAMs
IFN-TAMs
Reg-TAMs
Inflam-TAMs
Angio-TAMs
LA-TAMs
Baseline
G
Post-AI
H
Change

## Supplementary Files

This is a list of supplementary files associated with this preprint. Click to download.

- SupplementaryV4.pdf
